# The effect of fibrinoid necrosis on the clinical features and outcomes of primary IgA nephropathy

**DOI:** 10.1186/s12882-023-03419-4

**Published:** 2023-12-11

**Authors:** Hongshan Chen, Youxia Liu, Li Wei, He Wang, Zhenfeng Zheng, Tiekun Yan, Junya Jia, Dong Li

**Affiliations:** Department of Nephrology, Tianjin Medical University General Hospital, No. 154, Anshan Road, Heping District, Tianjin, PR China

**Keywords:** IgA nephropathy, Fibrinoid necrosis, Immunosuppressive therapy, Prognosis, The decline of eGFR

## Abstract

**Background:**

To explore the clinicopathologic features and outcomes of IgAN patients who presented with fibrinoid necrosis (FN) lesions or not and the effect of immunosuppressive (IS) treatment in IgAN patients with FN lesions as well.

**Methods:**

This was a retrospective cohort study with 665 patients diagnosed with primary IgAN from January 2010 to December 2020 in Tianjin Medical University General Hospital and having detailed baseline and follow-up characteristics. Patients were divided into two groups depending on the appearance of FN lesions. Patients with FN lesions were recruited into Group FN1, while patients who were not found FN lesions in their renal biopsy specimens were recruited into Group FN0. Compare the differences between Group FN0 and Group FN1 in baseline clinicopathologic features, treatment solutions and follow-up data as well. To evaluate the impact of different fractions of FN lesions on baseline characteristics and prognosis of IgAN, we subdivided patients in Group FN1 into 3 groups depending on the FN lesions distribution, Mild Group: 0 < FN% < 1/16; Moderate Group: 1/16 < FN% < 1/10; Severe Group: FN% > 1/10. Furthermore, we compared the differences in baseline clinicopathologic features, treatment solutions and follow-up data among these three groups. Kidney endpoint event was defined as patients went into end-stage kidney disease (ESKD), which estimated glomerular filtration rate (eGFR) < 15 ml/min/1.73 m^2, regularly chronic dialysis over 6 months or received renal transplantation surgery. The kidney composite endpoint was defined by a ≥ 30% reduction in eGFR, double Scr increase than on-set, ESKD, chronic dialysis over 6 months or renal transplantation. Compare the survival from a composite endpoint rate in different groups by Kaplan-Meier survival curve. The univariate and multivariate Cox models were used to establish the basic model for renal outcomes in patients with FN lesions.

**Results:**

(1) A total of 230 patients (34.59%) were found FN lesions in all participants. Patients with FN lesions suffered more severe hematuria than those without. On the hand of pathological characteristic, patients with FN lesions showed higher proportions of M1, E1, C1/C2 and T1/T2 lesions compared with those without FN lesions. (2) The 1-year, 3-year, and 5-year survival of the composite endpoint were lower in the FN1 group than FN0 group. (3) After adjusting for clinicopathological variables, the presence of FN lesions was a significantly independent risk factor for composite endpoint. By using multivariate Cox regression analyses, we also found when the fraction of FN lesions exceeded 10%, the risk of progression into composite endpoint increased 3.927 times.

**Conclusion:**

Fibrinoid necrosis of capillary loops is an independent risk factor of poor renal outcomes. More effective treatment should be considered for those who had FN lesions.

**Supplementary Information:**

The online version contains supplementary material available at 10.1186/s12882-023-03419-4.

## Introduction

IgA nephropathy (IgAN) is the most common type of primary glomerulonephritis worldwide especially in Asia and remains a leading cause of chronic kidney disease (CKD). Approximately 30 to 40% IgAN patients will develop into renal failure within 20–25 years from on-set [1]. Many studies were performed to evaluate the association between various risk factors, including proteinuria, level of glomerular filtration rate (GFR), hypertension and poor kidney outcomes [2–5]. Histologic data, The Oxford Classification of IgAN (MEST-C score) including mesangial hypercellularity (M), endocapillary hypercellularity (E), segmental glomerulosclerosis (S), interstitial fibrosis and tubular atrophy (T) and crescents (C), has been considered for the prognostication of IgAN [6, 7]. However, this calculation system did not consider all the pathological changes appearing in IgAN. The prevalence of other pathological characteristics occurred in IgAN still need more in-depth research.

Fibrinoid necrosis (FN) was defined as the fragmentation lesions in glomerular capillary characterized by the presence of fibrin-rich material around the capillaries. Over 6 to 15% IgAN biopsies would present FN lesions [8, 9]. A recent study made in Japan showed as an acute glomerular lesion, FN significantly correlated with the degree of daily proteinuria and hematuria [10]. FN was also reported as an active pathological finding in lupus nephritis, IgA vasculitis and anti-neutrophil cytoplasmic antibody (ANCA)- associated vasculitis and was sensitive to immunosuppressive treatment [11–13]. Whereas, whether FN lesions could be regard as a morphological indicator in the prognosis of IgAN is still unknown.

In this study, we analyze the clinicopathologic features and outcomes of IgAN patients who presented with FN lesions or not. In addition, we assessed the effect of immunosuppressive therapy in IgAN patients with FN lesions as well.

## Materials and methods

### Study design

**Fig. 1:**
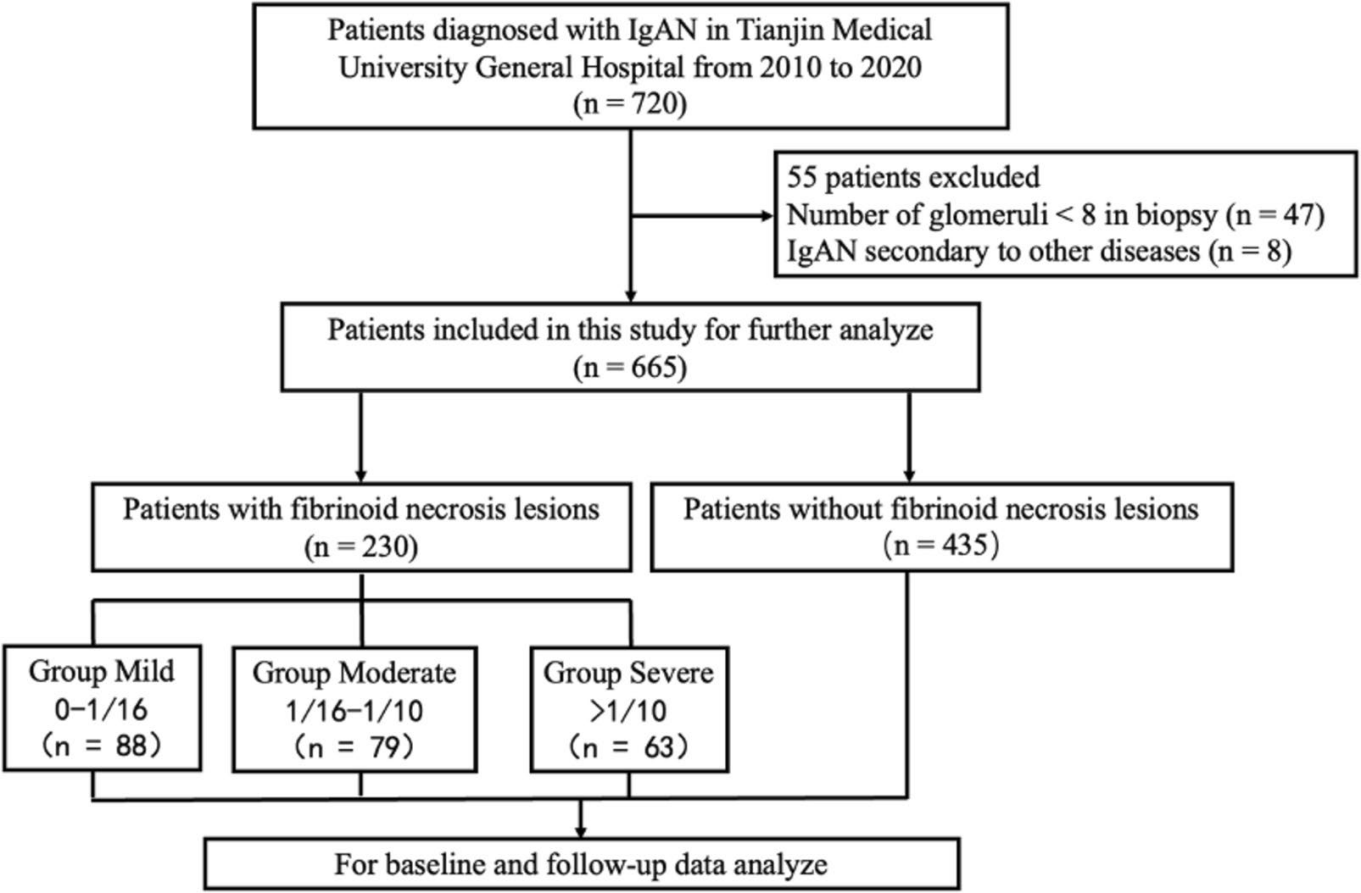
Flow diagram of the study population

This was a retrospective cohort study with patients diagnosed with primary IgAN from January 2010 to December 2020 in Tianjin Medical University General Hospital were enrolled in this study. Of these patients, 720 had detailed baseline and follow-up characteristics. Patients with IgAN secondary to liver diseases, systemic lupus erythematosus, Henoch-Schonlein purpura or IgAN with ANCA-associated glomerulonephritis as well as malignant tumor (*n* = 8) were excluded in our study. Patients with less than 8 glomeruli in biopsy specimens (*n* = 47) were also excluded. Finally, a total of 665 patients with IgAN were included in this study for baseline and follow-up data analysis (Fig. 1). Next, we roughly divide the FN1 group of patients into three equal parts based on the fraction of FN lesions to clarify the impact of FN lesion fraction on clinical manifestations and prognosis. Mild Group: 0 < FN% < 1/16; Moderate Group: 1/16 < FN% < 1/10; Severe Group: FN% > 1/10.

### Clinical data collection and definition

Demographic characteristics and clinical data including blood pressure (BP), mean arterial pressure (MAP), hemoglobin (Hb), serum albumin (Alb), serum creatinine (Scr), estimated glomerular filtration rate (eGFR), serum uric acid (UA), serum immunoglobulin A (IgA), proteinuria and hematuria were collected at the time of renal biopsy. MAP was defined as diastolic BP + 1/3 (systolic BP - diastolic BP). eGFR was calculated by the Chronic Kidney Epidemiology Collaboration (CKD-EPI) formula. The treatment solutions after renal biopsy were collected simultaneously. Renin-angiotensin system blocker (RASB) treatment was defined as the use of an angiotensin-converting enzyme inhibitor and/or angiotensin receptor blocker after renal biopsy. Immunosuppressive (IS) treatments referred to the use of glucocorticoid and/or immunosuppressive agents such as cyclophosphamide, mycophenolate mofetil, and cyclosporine regardless of dose. We recorded Scr, eGFR and proteinuria during follow-up.

### Pathological data collection and definition

All renal biopsy specimens were obtained by percutaneous needle biopsy and processed routinely for immunofluorescence microscopy, light microscopy and electron microscopy. All sections were stained with hematoxylin and eosin (HE), periodic acid-Schiff (PAS), PAS together with silver methenamine (PASM) and Masson’s trichrome. The biopsy specimens were reviewed independently by two renal pathologists using the updated Oxford Classification criteria. M1 was defined by the presence of more than three cells in mesangial area on more than half the glomeruli. E1 was defined as the appearance of an increased number of cells within capillary lumina causing narrowing of the lumina. S1 was defined as the appearance of tuft adhesion and segmental sclerosis but not global sclerosis. T was scored according to the estimated percentage of interstitial fibrosis and tubular atrophy in the cortex: T0 (25% of cortex), T1 (26–50%), T2 (> 50%). C was scored according to the fractions of glomeruli with cellular or fibrocellular crescents: C0 (absence of crescents), C1 (< 25%), C2 (25%). Fibrinoid necrosis (FN) lesions were defined as a segmental destructive lesion of the glomerular capillary loop, with fibrin deposition within and around the capillaries. FN0 referred to the patients without FN lesions and FN1 referred to the patients with FN lesions. Similar to the MEST-C scores, we tried to make a FN score which would have to be categorical and on the basis of one or more cutoffs that are easily determined. We reported the biopsy findings with a number of glomeruli with FN lesions and a total number of glomeruli, and the fraction of glomeruli with FN lesions was also measured stepwise.

### Outcome definition

The kidney composite endpoint was defined as the survival from a 30% reduction in eGFR, double Scr increase than on-set, end stage kidney disease (ESKD, eGFR < 15 ml/min/1.73 m^2), chronic dialysis over 6 months or renal transplantation.

### Statistical analyses

Normally distributed variables are described as means ± SD and compared using Student’s t test or one-way analysis of variance (ANOVA), nonparametric variables are described as medians [interquartile ranges] and compared using the Mann-Whitney U test or Kruskal-Wallis test. Categorical variables are described as percentages and compared using the chi-squared test. Person correlation test was carried out to test the correlation between FN lesions and the number of glomeruli. Interobserver agreement was investigated by calculating the κ value. Kaplan-Meier survival curve was used among Group Mild/Moderate/Severe vs Group FN0 and Group FN1 vs FN0 to explore the effects on the prognosis of kidney disease. The univariate and multivariate Cox models were used to establish the basic model for renal outcomes in patients with FN lesions. All *P* values were 2-tailed, and values < 0.05 were considered statistically significant. Statistical analyses were carried out using SPSS (version 26; IBM SPSS, Chicago, IL) and the R software (version 4.1.2; Free Software Foundation). Figures were produced by GraphPad Prism (version 9.0; GraphPad Software, USA).

## Results

### Clinical and pathological characteristics among patients with or without FN lesions

A total of 665 primary IgAN patients were enrolled in this study, and 230 of them had FN lesions. Initially, we tested the inter-rater reliability of FN lesions which showed a κ value of 0.73. Detailed clinical and pathological characteristics of IgAN patients with or without FN lesions were summarized in Table 1. Patients with FN lesions suffered more severe hematuria than those without. In addition, patients with FN lesions showed lower levels of Hb and serum Alb (126.9 ± 19.1 g/L vs 132.8 ± 19.7 g/L, *P* < 0.001; 36.7 ± 4.9 g/L vs 37.6 ± 5.7 g/L, *P* = 0.040). While, there was no significant difference in baseline renal function and proteinuria between two groups.

**Table 1:**
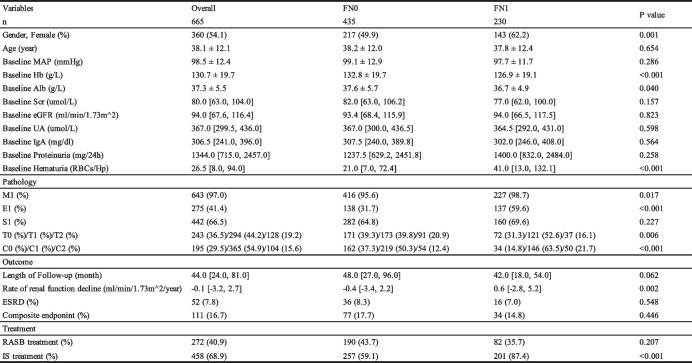
Clinical and pathological characteristics of patients with or without FN lesions in IgAN

*FN* fibrinoid necrosis lesions, *FN*0 patients without fibrinoid necrosis lesions, *FN1* patients with fibrinoid necrosis lesions, *MAP* mean arterial pressure, *Scr* serum creatinine, *eGFR* estimated glomerular filtration rate, *UA* serum uric acid, *MEST-C* MEST-C scores by Oxford classification, *ESRD* end stage renal disease, defined by eGFR < 15 ml/min/1.73 m^2; Composite endpoint, defined by either a ≥ 30% reduction in eGFR, a doubling of the base-line serum creatinine, ESRD, chronic dialysis for at least 6 months or renal transplantation, *RASB* renin-angiotensin system blocker, *IS* immunosuppressive agents

On the hand of pathological characteristic, patients with FN lesions showed higher proportions of M1, E1, C1/C2 and T1/T2 lesions compared with those without FN lesions (all *P* < 0.05).

The median length of follow-up was 44 months in the whole participants. The rate of renal function decline seems to be more slowly in FN1 groups than FN0 (0.6 [− 2.8,5.2] ml/min/1.73 m^2/year vs. -0.4 [− 3.4,2.2] ml/min/1.73 m^2/year, *P* = 0.002). The percentage of patients suffered from ESKD or composite endpoint showed no statistically significant in patients with or without FN lesions.

### Clinical and pathological characteristics among IgAN patients with different fractions of FN lesions

**Fig. 2:**
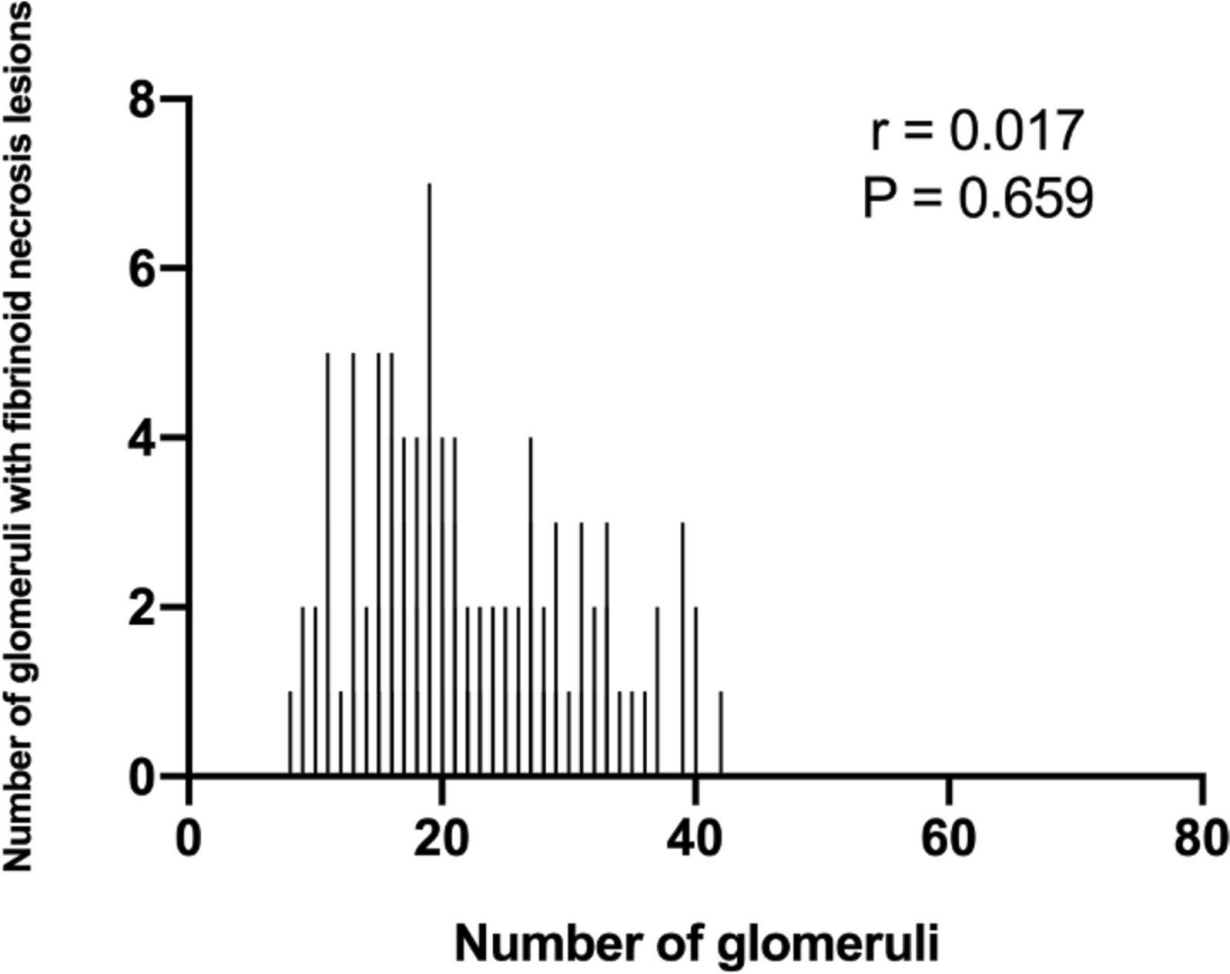
Correlation between the number of glomeruli and number of FN lesions

**Fig. 3:**
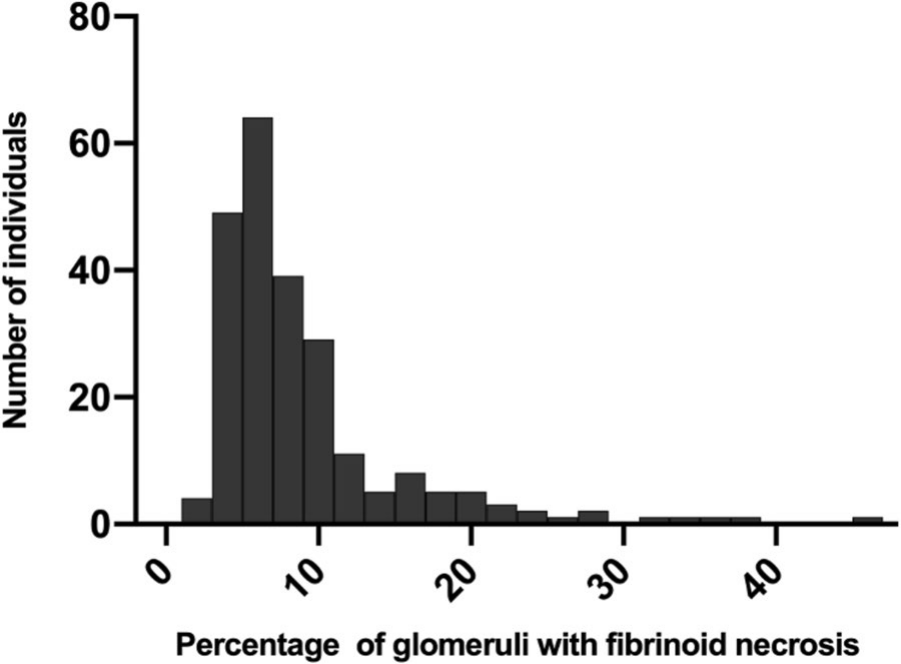
Distribution of the percentage of glomeruli with FN lesions in biopsies with any FN lesions

Due to the differences in clinical and pathological characteristics between IgAN patients with or without FN lesions, we next tried to find the impact of different fractions of FN lesions on baseline characteristics. Firstly, we found there was less correlation between the number of glomeruli and number of FN lesions (r = 0.017, *P* = 0.659, Fig. 2). Furthermore, the distribution of the percentage of glomeruli with FN lesions was shown in Fig. 3. Of the 230 patients with any faction of FN lesions, 74% had FN lesions less than 10%. We then divided the patients with FN lesions into three groups depending on the distribution. With the increase of the fraction with FN lesions, patients showed significantly decrease in eGFR (99.8 [76.0,121.4] ml/min/1.73 m^2 vs. 93.8 [66.0,111.5] ml/min/1.73 m^2 vs. 84.8 [60.7109.8] ml/min/1.73 m^2, *P* = 0.044), obviously increase in excretion in proteinuria (1100.0 [647.52104.5] mg/24 h vs. 1280.0 [888.0,1880.0] mg/24 h vs. 1690.5 [1192.53539.8] mg/24 h, *P* = 0.004, Table 2) and higher level of MAP (96.1 ± 10.6 mmHg vs. 96.5 ± 10.8 mmHg vs 101.2 ± 13.5 mmHg). Unlikely to baseline clinical characteristics, patients showed less difference in pathological changes with the increase of fraction of FN lesions. There was no difference in renal outcomes among these three groups. We found patients in the group with higher fraction of FN lesions were more likely to get immunosuppressive treatment (87.5% vs. 79.7% vs. 96.8%, *P* = 0.018).

**Table 2:**
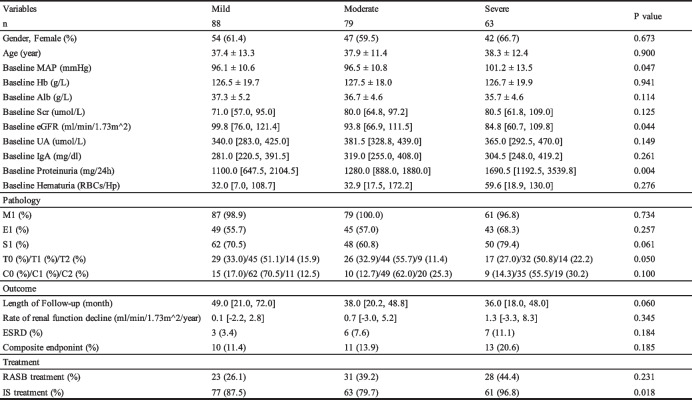
Clinical and pathological characteristics of patients with different fractions of FN lesions in IgAN

Mild represent the group of patients whose fraction of FN lesions is less than 1/16; Moderate represent the group of patients whose fraction of FN lesions is among 1/16 to 1/10; Severe represent the group of patients whose fraction of FN lesions is over 1/10, *MAP* mean arterial pressure, *Scr* serum creatinine, *eGFR* estimated glomerular filtration rate, *UA* serum uric acid, *MEST-C* MEST-C scores by Oxford classification; ESRD, end stage renal disease, defined by eGFR < 15 ml/min/1.73 m^2; Composite endpoint, defined by either a ≥ 30% reduction in eGFR, a doubling of the base-line serum creatinine, ESRD, chronic dialysis for at least 6 months or renal transplantation, *RASB* renin-angiotensin system blocker, *IS* immunosuppressive agents

### IS treatment in IgAN patients with or without FN lesions

**Fig. 4:**
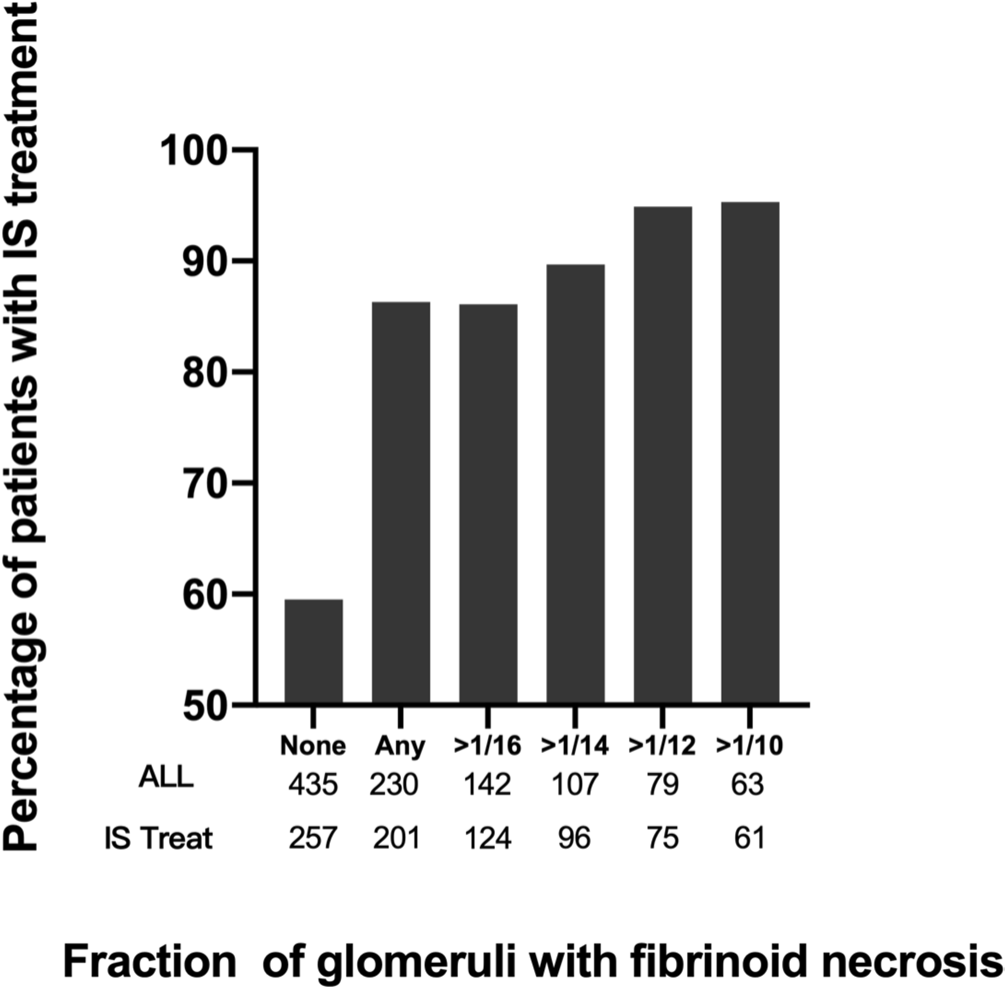
Association between the presence and fraction of glomeruli with FN lesions and the subsequent use of IS

As shown in Table 1, patients with FN lesions were more likely to receive IS treatment (FN1 vs FN0, 87.4% vs 59.1%, *P* < 0.001). We further evaluated the subsequent use of IS agents in different fraction of FN lesions. 87.4% of patients with any fraction of FN lesions received IS treatment compared with 59.1% of patients without FN lesions. Stepwise examination of increasing fractions of glomeruli with FN lesions revealed a steady increase in the percentage of patients treated with immunosuppression agents (Fig. 4).

### Kidney outcome and adjusted predictive value of FN lesions

**Fig. 5:**
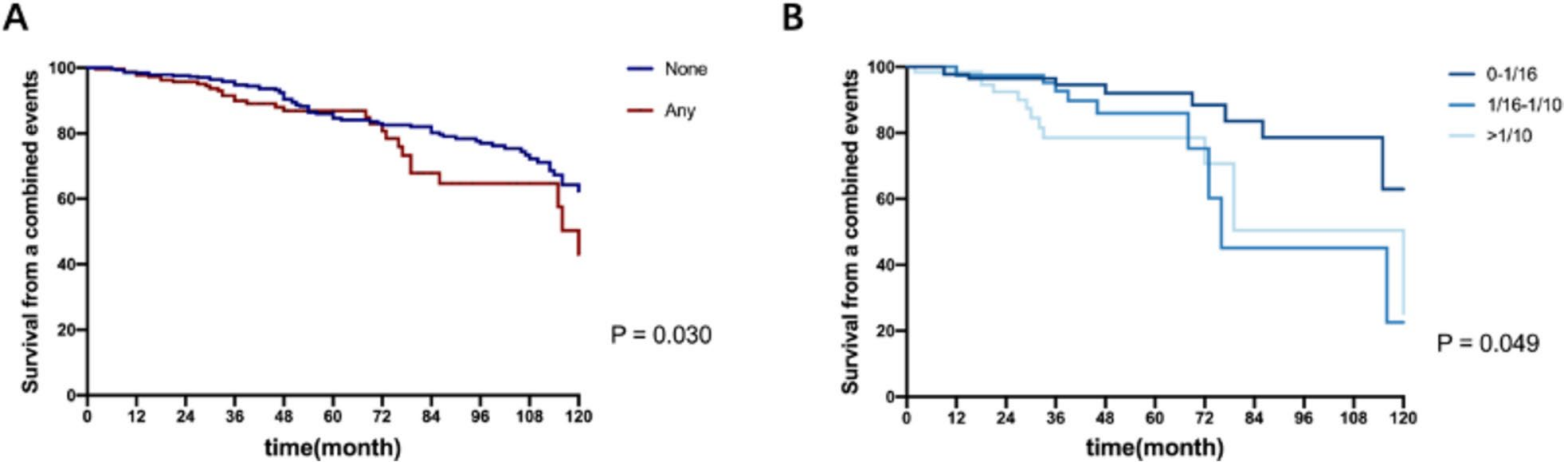
Univariate effect of the presence and fraction of glomeruli with FN on survival from a composite event. **A** Kaplan-Meier survival curves of patients with FN lesions or not surviving from a composite endpoint. **B** Kaplan-Meier survival curves of patients with different fractions of FN lesions surviving from a composite endpoint

**Fig. 6:**
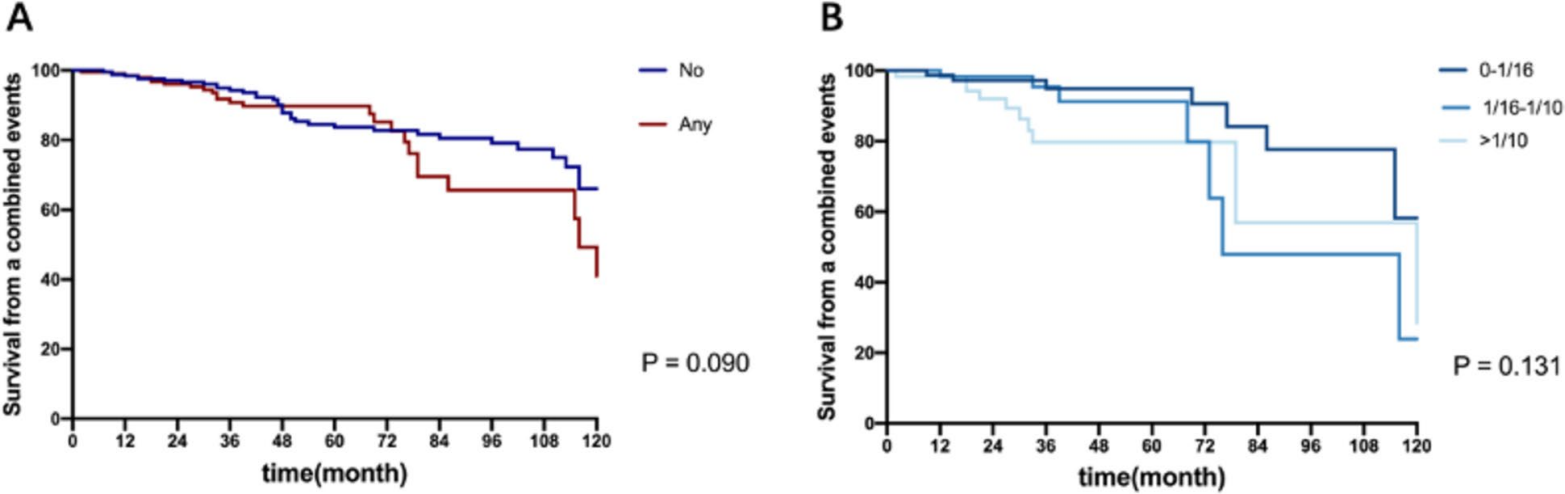
Univariate effect of the presence and fraction of glomeruli with FN on survival from a composite event in IS group. **A** Kaplan-Meier survival curves of patients with FN lesions or not surviving from a composite endpoint after IS treatment. **B** Kaplan-Meier survival curves of patients with different fractions of FN lesions surviving from a composite endpoint after IS treatment

A total of 111 patients (16.7% of all patients) reached composite kidney endpoint. The 1-year, 3-year, and 5-year survival of the composite endpoint were 91.8, 49.4, 20.2% in the FN group and 95.8, 64.8, 38.4% in the no-FN group (Fig. 5). These differences were statistically significant (all *P* < 0.050). By contrast, patients showed no statistical significance on the survival of the composite kidney endpoint with or without FN lesions after IS treatment (Fig. 6).

**Fig. 7:**
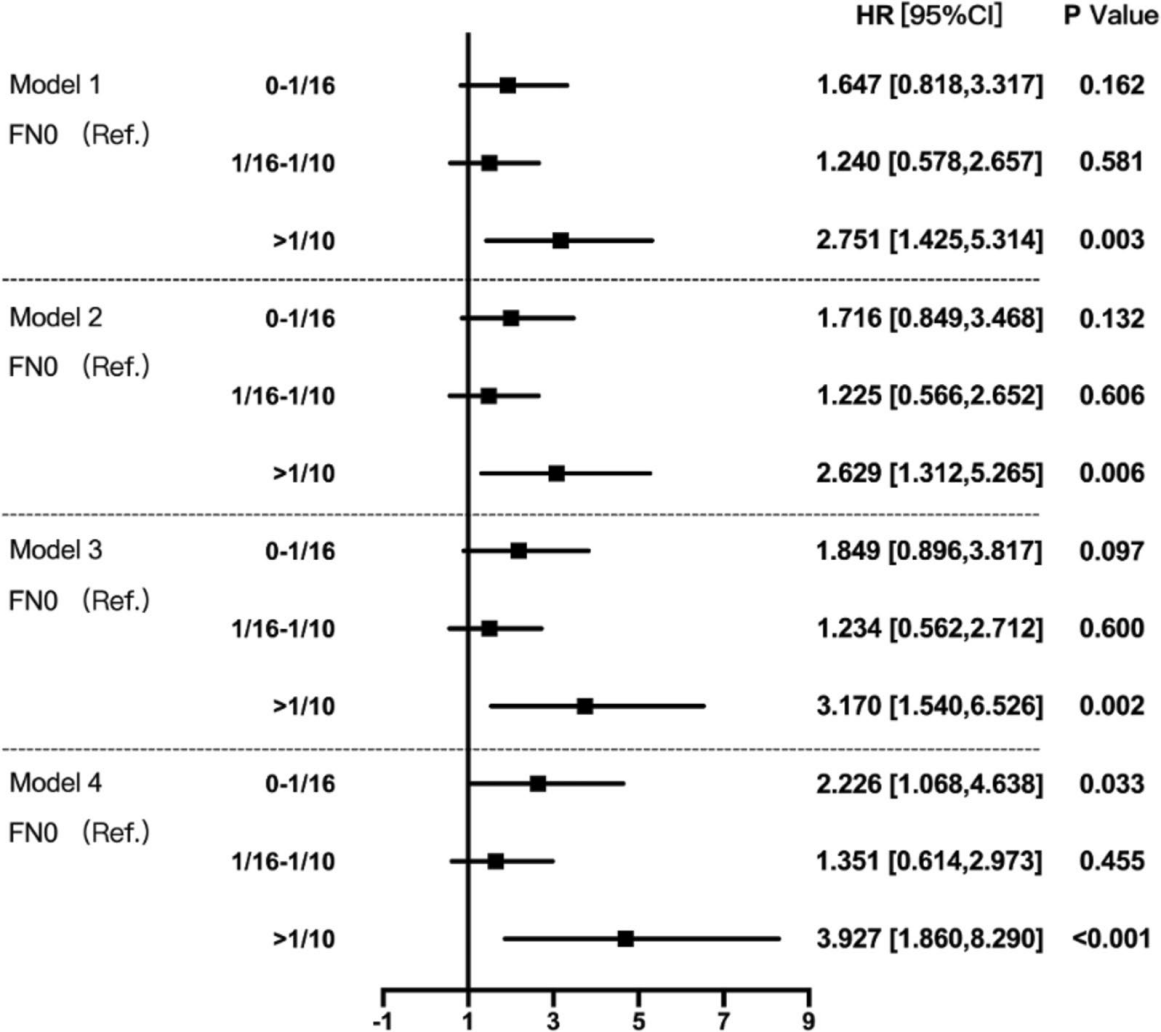
Multivariate effect of the fraction of glomeruli with FN lesions on survival from combined event. Model 1: adjusted for eGFR and proteinuria; Model 2: adjusted for Model 1 plus Hb and Alb; Model 3: adjusted for Model 2 plus METC lesions; Model 4: adjusted for Model 3 plus IS treatment

Univariate analyses followed by multivariate analyses were carried out to examine the predictive value of FN lesions for kidney outcome. Clinicopathological parameters were used in the univariate analyses: gender, baseline Hb and Alb, initial eGFR, proteinuria, hematuria, treatment modalities, MEST-C scores based on the updated Oxford classification and FN lesions. In univariate Cox analyses, FN lesions whatever fraction (HR 1.567, 95% CI 1.041–2.360, *P* = 0.032), as well as Hb, Alb, eGFR, proteinuria, mesangial hypercellularity, endocapillary hypercellularity, tubular atrophy/interstitial fibrosis and crescents, were strongly associated with renal survival (Table 3). Moreover, after adjustment for all model factors, FN lesion was still an independent predictor for renal survival in multivariate Cox analyses (Table 4, Supplemental Table 1). To evaluate the effect of the FN fractions on the prognosis of IgAN, we carry out univariate and multivariate COX analyses on the FN fractions as well. As shown in Fig. 7, after adjustment for all model factors, when IgAN patients had a fraction of FN more than 1/10, the risk to reaching a composite endpoint would increase 3.927 folds (VIF 1.144, *P* < 0.001).

**Table 3:**
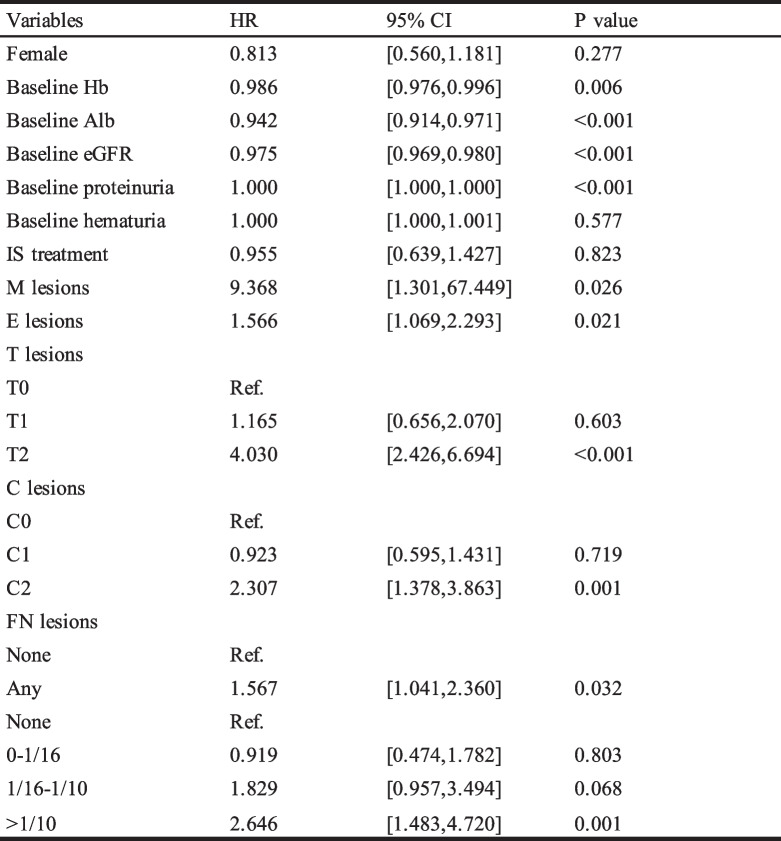
Univariate determinants of survival from a combined event

*eGFR* estimated glomerular filtration rate, MEST-C MEST-C scores by Oxford classification, *IS* immunosuppressive agents, *FN* fibrinoid necrosis lesions

**Table 4:**
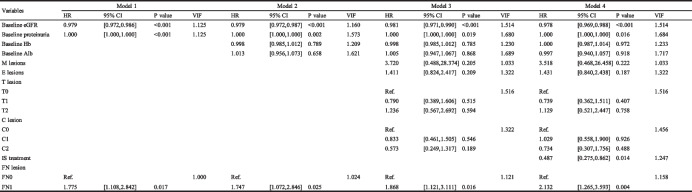
Multivariate effect of the presence of glomeruli with FN lesions on survival from combined event

Model 1: adjusted for eGFR and proteinuria; Model 2: adjusted for Model 1 plus Hb and Alb; Model 3: adjusted for Model 2 plus METC lesions; Model 4: adjusted for Model 3 plus IS treatment

In order to further investigate the impact of immunosuppressive therapy on the prognosis of IgA nephropathy (IgAN) patients with concurrent FN lesions, a multivariable Cox regression analysis was performed on the population treated with immunosuppressive therapy. As shown in Table 5, after adjusting for relevant clinical, pathological, and treatment-related factors, FN lesions remained an independent risk factor for composite endpoint events in IgAN (HR 2.099, *P* = 0.020). As shown in Table 6, in the multivariable Cox regression model adjusted for clinical and pathological influencing factors, an FN proportion exceeding 1/10 was still an independent risk factor for the occurrence of kidney composite endpoint events (HR 3.397, *P* = 0.004).

**Table 5:**
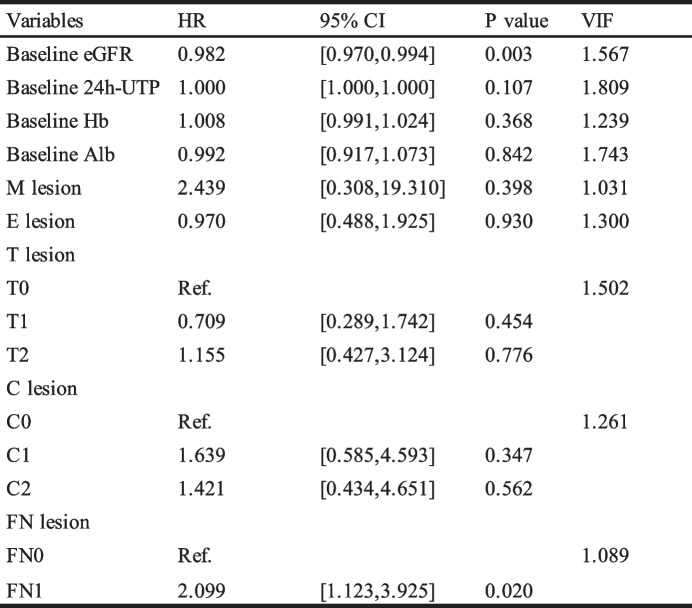
Multivariate effect of the presence of glomeruli with FN lesions on survival from combined event in IS group

**Table 6:**
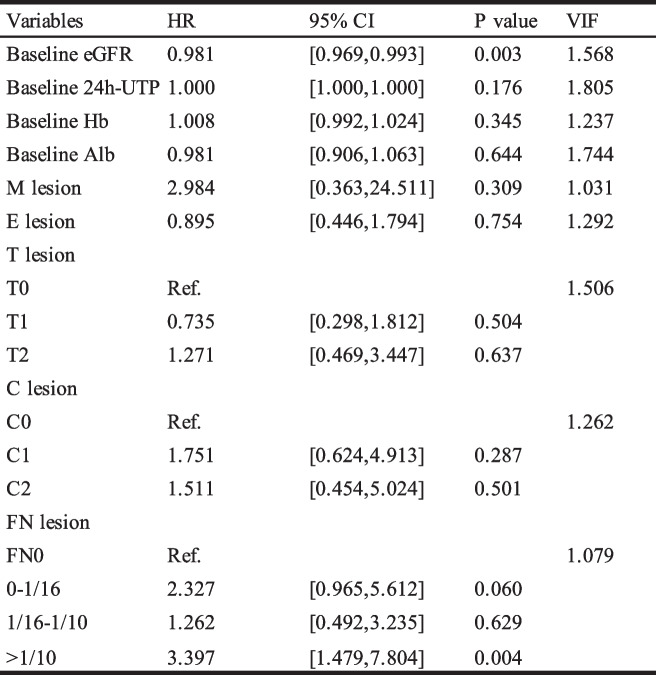
Multivariate effect of the fraction of glomeruli with FN lesions on survival from combined event in IS group

## Discussion

IgAN is a common complex disease with multiple factors involved in disease development and progression. As a rare phenomenon in IgAN, few studies reported the effect of FN lesions on the kidney function decline. In our study, 230 of 665 (34.59%) patients with IgAN were found fibrinoid necrosis lesions regardless of the fraction, of which 74% had a fraction of glomeruli with FN lesions less than 10%. We also found that there was little correlation of between the number of glomeruli and FN lesions, which differed from previous reported [14]. Furthermore, we found patients with fibrinoid necrosis had a higher risk to suffer renal function decline than those without, though there is little difference in clinical characteristics except the severity of hematuria between these two group patients. Immunosuppressive therapy seems to provide little benefit effect to patients with FN lesions. Based on these findings, we conjectured FN lesions might be a rare but an important indicator of prognosis and disease activity of IgAN.

FN is limited to small blood vessels including small arteries, arterioles, and glomeruli [15]. Fibrinoid necrosis of arteries is linked to endothelial damage and is characterized by the infiltration and accumulation of serum proteins, which are then followed by fibrin polymerization within the vessel wall. These substances combine to create a highly eosinophilic collar that obscures cellular features. This condition is common in various acute degenerative and inflammatory disorders of small arteries and arterioles [16]. Furthermore, our comparison of patients with and without FN lesions showed that the patients in FN group suffered heavier hematuria and had greater Oxford pathologic scores. This result is consistent with Taiko et al. who demonstrated that fibrinoid necrosis representing the acute glomerular pathological lesion was correlated significantly with the degree of hematuria [10]. R. Tewari et al. also showed that endocapillary proliferation and other renal vasculitic lesions could be thought as histological parameters which showed close association with hematuria [17]. While, when comparing other clinical features especially the severity of proteinuria and renal dysfunction no significant difference was found between patients with or without FN lesions. It may be because most patients in FN group have a lower fraction of glomerular with FN lesions. To further validate the difference in patients with different fractions of FN lesions, we divided patients with FN lesions into three groups according to the distribution of FN lesions fraction. It is obvious to see that with the increase of FN lesions fraction, patients revealed heavier load of proteinuria and lower level of eGFR.

The prognosis for patients with IgAN is frequently poor, with 25–30% of them developing progressive renal failure within 20–25 years after initial diagnosis and an estimated 1–2% of adults developing ESRD each year [18, 19]. However, the significance of FN lesions for the prognosis of IgAN is still unclear. In this study, we found the decline of eGFR in patients with FN lesions was slower than that in patients without FN lesions, which might be the result of increased use of immunosuppressive therapy in patients with FN lesions. After adjusting for clinicopathological variables, the presence of FN lesions was a significantly independent risk factor for composite endpoint. By using multivariate Cox regression analyses, we also found when the fraction of FN lesions is more than 10%, the risk of progression into composite endpoint increases.

As reported by D’Amico et al. Thirty Five out of 340 patients (10.3%) biopsied in the study showed active segmental necrotizing lesions [16]. Moreover, a study carried out by Japanese researchers revealed that through continuous biopsy observation of renal biopsy specimens the incidence of fibrinoid necrosis of capillary loops also increased with the increase of section level, up to 30% [20]. All of the above findings suggested that FN is a quite common phenomenon in IgAN. It occurs when an autoimmune inflammatory disease (e.g., ANCA-associated vasculitis or lupus nephritis) or damage blood vessels. T. Zhang et al. validated that M2 macrophages may play an important role in driving or modulating interstitial inflammation, cellular crescent formation, and fibrinoid necrosis, and the expression of CD163 which is the specific marker for activated M2 macrophages has been reported to be influenced by several immunosuppressive medications [13, 21]. Interestingly, M2 macrophages were positively correlated with fibrotic area which has been found in IgAN patients [22]. However, whether FN lesion is an indicator for immunosuppressive therapy in IgAN is still lack of evidence. In our present study, multivariable Cox regression analysis, whether in all IgAN patients or in IgAN patients treated with immunosuppressive therapy, suggests that even after adjusting for the kidney protective effects of immunosuppressive therapy on IgAN, the presence and increasing fraction of FN lesions remain independent risk factors for adverse renal outcomes in IgAN. Adequate anti-inflammation and immunosuppression cannot ameliorate the renal damage caused by FN lesions. Understanding the mechanisms behind FN lesion occurrence and developing more targeted and effective treatments are of paramount importance for improving the prognosis of IgAN patients with concurrent FN lesions.

Several limitations of this study should be acknowledged. First, this was a retrospective cohort study and the patients were all from a single hospital in China. Thus, the sample size and the strategy of treatment may not be uniform, which may lead to some bias. Second, some patients without detailed follow-up information were excluded in our study and the length of follow-up time may not be long enough. Finally, the number of patients who did not receive IS treatment is rare which means we could not explore the value separately. A multi-center prospective randomized controlled studied should be carried out to confirm these contradictory results in the future.

## Conclusion

In conclusion, we found fibrinoid necrosis of capillary loops is an independent risk factor of poor renal outcomes. More effective treatment should be considered for those who had FN lesions.

### Supplementary Information

**Additional file 1: Supplemental Table 1. **Multivariate effect of the presence of glomeruli with FN lesions on survival from combined event. Supplemental Table 2. Multivariate effect of the fraction of glomeruli with FN lesions on survival from combined event.

## Data Availability

Raw data used during the current study are available from the corresponding author on reasonable request for non-commercial use.
