# Supplementary material for: The effect of fibrinoid necrosis on the clinical features and outcomes of primary IgA nephropathy
Source: BMC Nephrol. 2023 Dec 11;24:366. doi: 10.1186/s12882-023-03419-4 (PMC10712095; doi:10.1186/s12882-023-03419-4)

**Supplemental Table 1** **Multivariate effect of the presence of glomeruli with FN lesions on survival from combined event.**


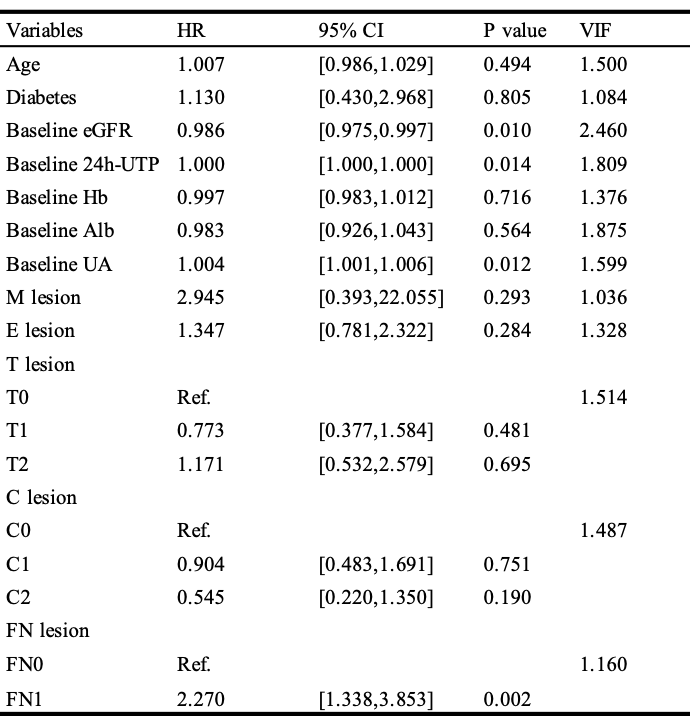


**Supplemental Table 2 Multivariate effect of the fraction of glomeruli with FN lesions on survival from combined event.**


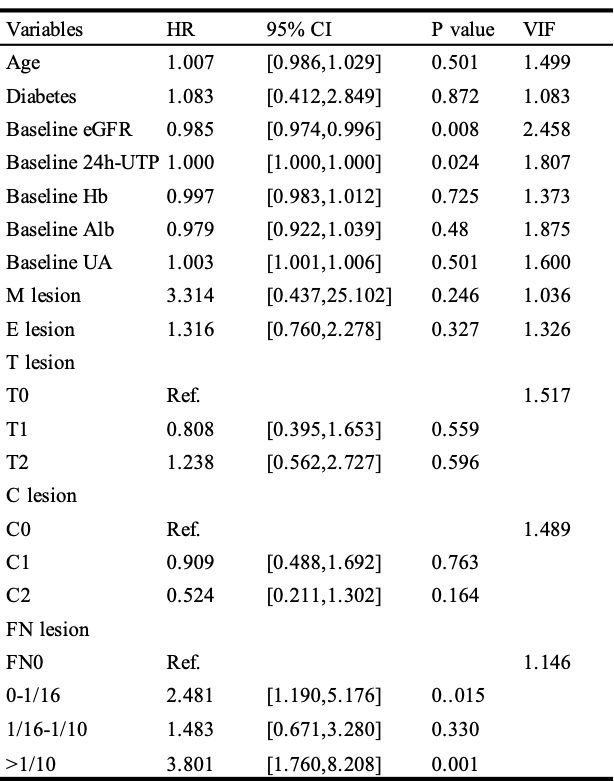

Supplement: Supplementary file 1 — Additional file 1: Supplemental Table 1. Multivariate effect of the presence of glomeruli with FN lesions on survival from combined event. Supplemental Table 2. Multivariate effect of the fraction of glomeruli with FN lesions on survival from combined event. [file 12882_2023_3419_MOESM1_ESM.docx]
